# Gold nanoparticles deposited on glass: physicochemical characterization and cytocompatibility

**DOI:** 10.1186/1556-276X-8-252

**Published:** 2013-05-25

**Authors:** Alena Reznickova, Zdenka Novotna, Nikola Slepickova Kasalkova, Vaclav Svorcik

**Affiliations:** 1 Department of Solid State Engineering, Institute of Chemical Technology, Prague 166 28, Czech Republic

**Keywords:** Glass, Gold sputtering, Gold nanoparticles, Surface properties, Cell adhesion and proliferation

## Abstract

Properties of gold films sputtered under different conditions onto borosilicate glass substrate were studied. Mean thickness of sputtered gold film was measured by gravimetry, and film contact angle was determined by goniometry. Surface morphology was examined by atomic force microscopy, and electrical sheet resistance was determined by two-point technique. The samples were seeded with rat vascular smooth muscle cells, and their adhesion and proliferation were studied. Gold depositions lead to dramatical changes in the surface morphology and roughness in comparison to pristine substrate. For sputtered gold structures, the rapid decline of the sheet resistance appears on structures deposited for the times above 100 s. The thickness of deposited gold nanoparticles/layer is an increasing function of sputtering time and current. AFM images prove the creation of separated gold islands in the initial deposition phase and a continuous gold coverage for longer deposition times. Gold deposition has a positive effect on the proliferation of vascular smooth muscle cells. Largest number of cells was observed on sample sputtered with gold for 20 s and at the discharge current of 40 mA. This sample exhibits lowest contact angle, low relative roughness, and only mild increase of electrical conductivity.

## Background

Gold nanoparticles (GNPs) are currently used as catalysts [1], and chemical [2] and plasmonic sensors [3]. They are also used in surface-enhanced Raman scattering [4] and nonlinear optics [5]. Furthermore, the usage of GNP for diagnosis and even destruction of microorganisms [6] or AuNP for biological applications [7-9] should be mentioned. Although GNPs are believed to be biologically inert, they can be engineered to possess chemical and biological functionality. GNP exhibits a plasmon resonance (PR) at wavelengths from 510 to 580 nm [10] leading to enhanced absorption and scattering in this part of the optical spectrum. The PR is affected by the size and shape of the GNP, the type of the supporting substrate (mainly its refractive index) and/or the surrounding material of the gold nanoparticles. The distance between the nanoparticles is also relevant, especially if it is small enough to enable electromagnetic coupling [11]. GNPs are usually prepared by precipitation from aqueous solutions [12,13] on various materials, e.g., on etched glass surfaces [13,14]. Thermal annealing of thin gold films produced by evaporation or sputtering [15] can also lead to a gold aggregation into GNP [16]. The formation of GNP from continuous gold layers is driven by the minimization of the surface energy and is denoted as solid state dewetting [17]. However, all the described methods suffer from the poor adhesion of GNP to the substrate surface [18].

It is known that the biocompatibility of a substrate is affected, besides of several other factors, by their electrical conductivity, chemical structure, surface morphology, roughness, and wettability (polarity) [19]. In this work, we studied the surface morphology, sheet electrical resistance, contact angle, ultraviolet–visible (UV–vis) spectra, adhesion, and proliferation of living muscle cells on gold structure sputtered on glass surface.

## Methods

### Materials and modification

The gold layers were sputtered on 1.8 × 1.8 cm^2^ microscopic glass, supplied by Glassbel Ltd., Prague, Czech Republic. The surface roughness of glass, measured over the area of 1×1 μm^2^ and calculated as an average value from five different measuring positions, was *R*_*a*_ = 0*.*34 ± 0*.*03 nm [16]. The gold sputtering was accomplished on Balzers SCD 050 device from gold target (supplied by Goodfellow Ltd., Huntingdon, England). The deposition conditions were DC Ar plasma, gas purity of 99.995%, sputtering time of 10 to 400 s, current of 10 to 40 mA (discharge power 3 to 15 W), total Ar pressure about 5 Pa, and the electrode distance of 50 mm. The power density of Ar plasma in our case was 0.13 W·cm^−2^, and the average deposition rate was 0.15 nm s^−1^. The glass substrate was cleaned with methanol (p.a.) and dried in a stream of N_2_. The prepared samples were stored at laboratory conditions.

### Measurement techniques

The mean thickness of gold films was measured by gravimetry using Mettler Toledo UMX2 microbalance (Columbus, OH, USA). The thickness was calculated from the sample weights before and after sputtering using gold bulk density.

The sheet resistance of Au layers was examined by Ohm’s method with a picoampermeter Keithley487 (Cleveland, OH, USA). For the measurement, two Au contacts, about 50-nm thick, were deposited on the layer surface by sputtering. The samples with lower resistances (up to 1 MΩ) were measured on the commercially available multimeter UNI-T 83 (Uni-Trend Group Limited, Kowloon, Hong Kong). The electrical measurements were performed at a pressure of about 10 Pa to minimize the influence of atmospheric humidity. The typical error of the sheet resistance measurement did not exceed ±5%.

Static contact angles (CA) of distilled water, characterizing structural and compositional changes caused by the gold deposition, were measured at room temperature at two samples and at seven positions using a Surface Energy Evolution System (SEES, Masaryk University, Brno, Czech Republic). Drops of 8.0 ± 0.2 μl volume were deposited using automatic pipette (Transferpette Electronic Brand, Wertheim, Germany), and their images were taken with 5-s delay. Then, the contact angles were evaluated using the SEES code.

UV–vis absorption spectra were recorded using a Varian Cary 25 Scan UV–vis spectrophotometer (PerkinElmer Inc., Waltham, MA, USA). UV–vis spectra in the range from 300 to 900 nm were taken with 1-nm data step at the scan rate of 240 nm·min^−1^. The results are presented as difference spectra (delta absorbance) obtained by the substraction of reference spectrum of pristine glass from the spectra of sputtered samples.

The surface morphology of glass and gold-sputtered glass was examined by atomic force microscopy (AFM) using VEECO CP II setup (phase mode);the surface roughness (*R*_a_) was measured in taping mode (Bruker Corp., Madison, WI, USA). Si probe RTESPA-CP with the spring constant 0.9 N m^−1^ was used. By the repeated measurements of the same region (1 × 1 μm^2^ in area), we prove that the surface morphology did not change after three consecutive scans.

### Cell culture, adhesion, and proliferation

For the study of cell adhesion and proliferation of six samples, gold coated under different conditions, were used. The glass samples were sterilized for 1 h in ethanol (75%), air-dried, inserted into polystyrene 12-well plates (TPP, Trasadingen, Switzerland; well diameter 20 mm), and seeded with vascular smooth muscle cells (VSMCs) derived from the rat aorta using an explantation method [20]. VSMCs were seeded on the samples with the density of 16,000 cells·cm^−2^ into 3 ml of Dulbecco’s modified Eagle’s minimum essential medium (Sigma, USA, cat. no. D5648), containing 10% fetal bovine serum (Sebak GmbH, Aidenbach, Germany). Cells were cultivated at 37°C in a humidified air atmosphere containing 5% of CO_2_. The number and the morphology of initially adhered cells were evaluated 24 h after seeding. The cell proliferation activity was estimated from the increase in the cell numbers achieved on the 3rd and 6th days after seeding [9]. The number and the morphology of the cells on the sample surface were then evaluated on microphotographs taken under an Olympus IX 51 microscope (Waltham, MA, USA; objective of 20×, visualized area of 0.136 mm^2^), equipped with an Olympus DP 70 digital camera. The number of the cells was determined using the image analysis software NIS-Elements (Melville, NY, USA). For each sample type, 20 independent measurements were performed. The number of adhered and proliferated cells was determined from the six samples. One sample of the particular type was used for the determination of the viability of the cells [9]. The determination of cell viability was accomplished on cell viability analyzer (Vi-CELL XR, Beckman Coulter, Fullerton, CA, USA) using elimination test with trypanose blue. This color penetrates through the cell membrane into the dead or damaged cells and accumulates inside. The living cells are not colored. On the base of different coloration, the numbers of living and dead cells are determined, and their viability is evaluated.

## Results and discussion

**Figure 1:**
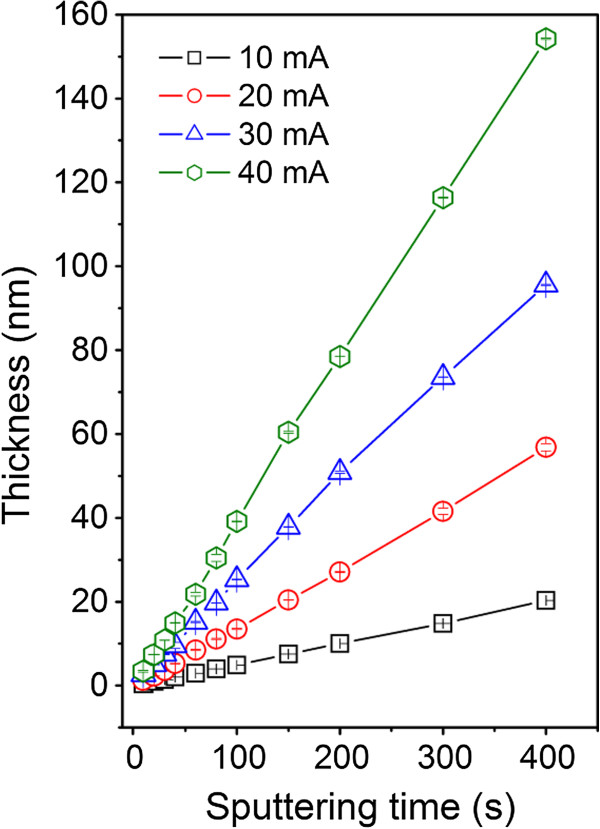
**Dependence of the thicknesses of gold film sputtered on glass.** On sputtering time at discharge currents 10, 20, 30, and 40 mA.

The thickness of the gold layers as a function of the sputtering time and discharge current, determined from gravimetry, is shown in Figure 1. Linear dependence between the sputtering time and the layer thickness is evident even in the initiatory stage of the layer growth. As could be expected, the film thickness is an increasing function of the sputtering time and discharge current as well. The dependence on the discharge current is not linear but closer to quadratic one. For 400-s deposition time, the film thicknesses are 20, 58, 95, and 155 nm for the discharge currents 10, 20, 30, and 40 mA, respectively.

**Figure 2:**
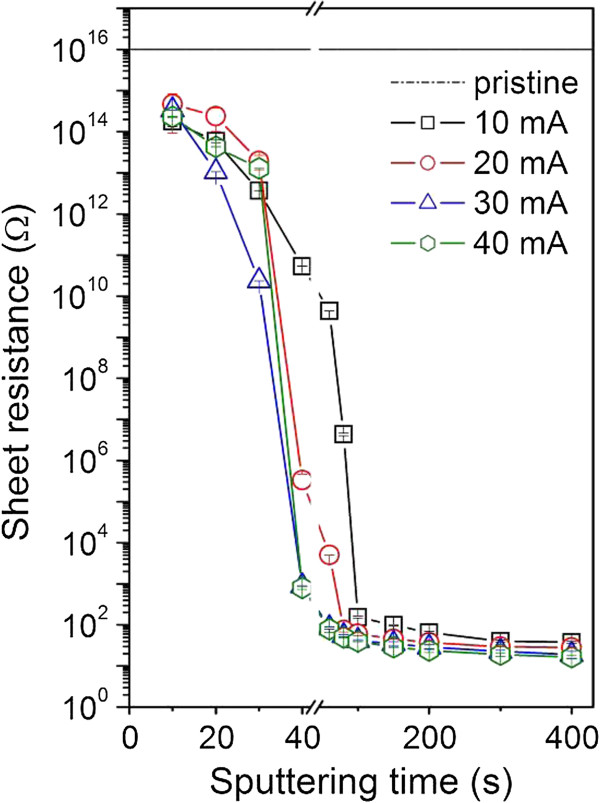
Dependence of the sheet resistance of the gold film on the sputtering time and on discharge current.

Figure 2 shows the dependence of the electrical sheet resistance of the gold films on the sputtering time for different sputtering currents. It is well known that a rapid decline of sheet resistance of sputtered layers indicates transition from discontinuous to continuous gold coverage [21]. One can see that the most pronounced change in the sheet resistance occurs in the sputtering time interval from 20 to 60 s. After a continuous coverage is formed, the sheet resistance decreases rapidly. The resistance of thin gold film, deposited for e.g., 100 s, is higher in comparison with that of the bulk gold due to the size effect in accord with the Mattheissen rule [22]. One can see that the layer resistances are about one order of magnitude higher than that reported for the metallic bulk gold (*R*_Au_=2.5 × 10^−6^Ω cm) [23]. One can also see that the resistance is a decreasing function of the discharge current.

**Figure 3:**
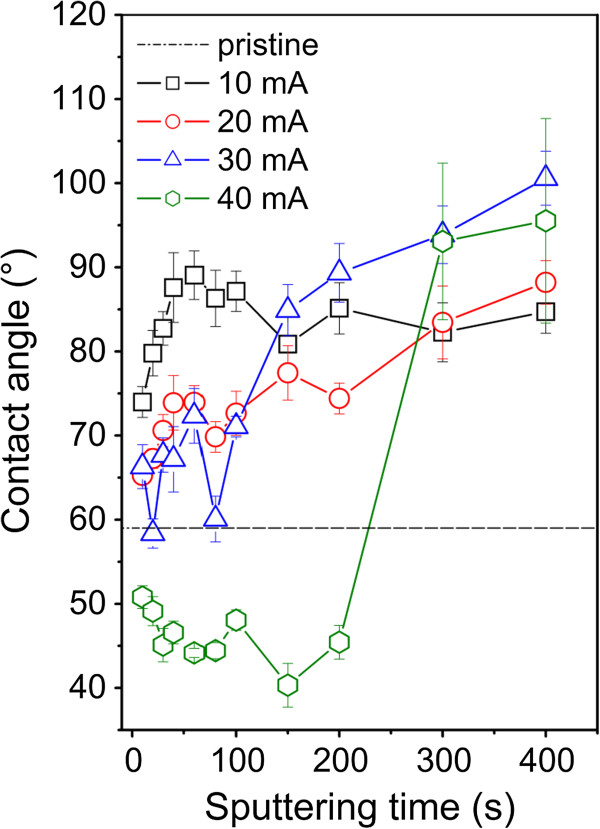
Dependence of the contact angle on the sputtering time and on discharge current.

Substrate biocompatibility is affected by the surface wettability (surface polarity). Gold-coated surfaces are expected to be more hydrophobic. These phenomena can be explained by charge-inducing mechanism at metal-solution interface caused by electron accumulation [24]. In Figure 3, the dependence of the CA on the sputtering time and discharge current for gold-coated glass are shown. The contact angle is a slowly increasing function of the sputtering time for discharge currents from 10 to 30 mA. Initial irregularities in the dependence may be due to the creation of isolated gold islands of different sizes and densities. After the formation of continuous gold coverage, the samples exhibit hydrophobic character [24]. Dramatically different dependences of CA on the sputtering time for the sputtering times below 200 s exhibit samples sputtered at the 40-mA discharge current. In this case, the gold-sputtered samples have CA lower than that of the pristine glass.

**Figure 4:**
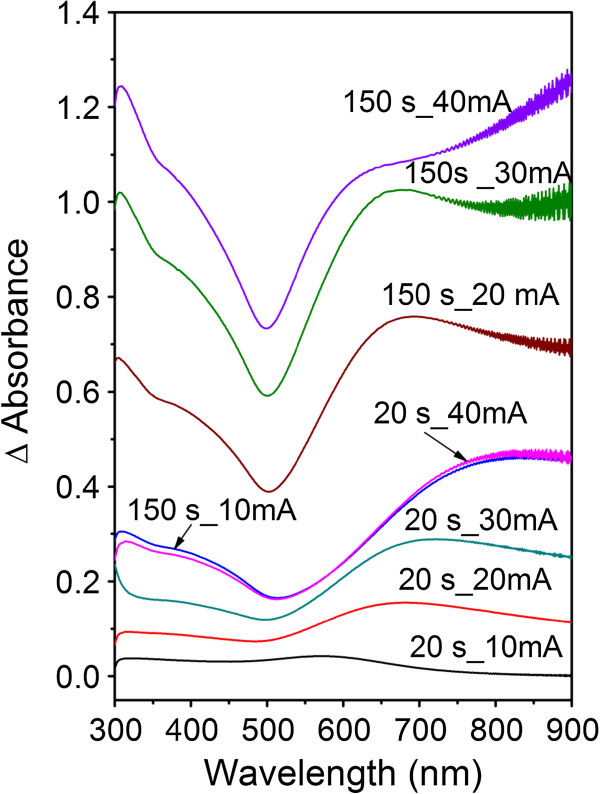
**UV–vis spectra of gold films deposited on glass.** Sputtering times 20 and 150 s and discharge currents 10, 20, 30, and 40 mA.

Thin Au films exhibit structure-dependent UV–vis optical spectra [21]. The delta absorption UV–vis spectra of the samples which are gold sputtered for the sputtering times 20 and 150 s at the discharge currents from 10 to 40 mA is shown in Figure 4. The absorbance of gold structures increase with increasing sputtering time and discharge current and film thickness as could be expected. Discontinuous and inhomogeneous layers are composed of nanometer-sized gold particles. It is well known that the optical absorption of the structures composed of gold islands is a function of island size and density [25]. On the UV–vis spectra, the broadband of plasmon resonance, situated at about 500 nm, is clearly visible. The band is more pronounced on the samples sputtered for longer times and at higher discharge currents.

**Figure 5:**
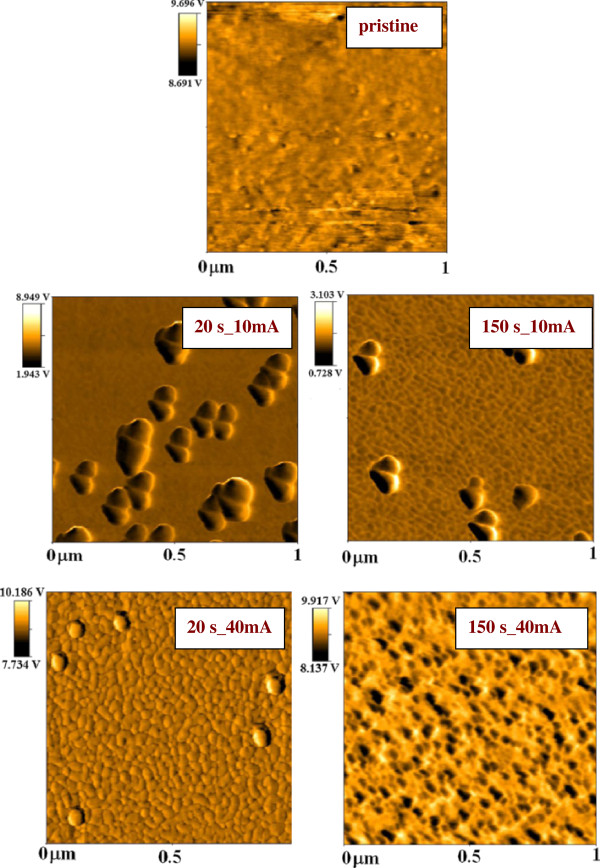
**AFM images (taken in phase mode) of pristine glass and gold-coated glass.** Sputtering times 20 and 150s and currents 10 and 40 mA.

The 2-D AFM images taken in phase mode on pristine glass and selected gold-coated samples are shown in Figure 5. On the sample sputtered for 20 s at the discharge current of 10 mA, the isolated gold islands are clearly visible. After the 150-s sputtering time at the same current, electrically continuous gold film is formed (see also Figure 2). On the samples sputtered at the discharge current of 40 mA for 20/150 s, electrically discontinuous/continuous gold film is formed [26] as can be seen from the AFM images too.

The surface roughness *R*_a_ of glass with gold film sputtered for different sputtering times and discharge currents are summarized in Table 1. Surface roughness of glass is *R*_a_ = 0*.*34 nm. As could be expected, the gold coverage leads to an increase of the surface roughness. Both the samples with discontinuous and continuous gold coverage were chosen for comparison. It is evident that the roughness is affected by several factors such as degree of gold coverage and the size and height of gold islands in the case of incomplete coverage, but no clear trend in the roughness dependence can be identified. Only some partial conclusions can be made. For example, the sample sputtered for 20 s at 10 mA exhibits higher *R*_a_ than those sputtered for the same time at 20 and 30 mA. The difference may be caused by larger number of high isolated gold islands created by nucleation at lower discharge current.

**Table 1:** **Surface roughness *R***_**a **_**of glass with gold film sputtered for different sputtering times and discharge currents**

	**Surface roughness (nm)**			
**Sample**	**10 mA**	**20 mA**	**30 mA**	**40 mA**
Glass/Au (20 s)	3.8	1.3	0.5	1.2
Glass/Au (150 s)	1.1	5.3	2.0	1.6

The surface roughness *R*_a_ of pristine glass and glass with gold film sputtered for different sputtering times (20 s, 150 s) and discharge currents (10 to 40 mA). Pristine glass has *R*_a_= 0.34 nm. Typical error is ±10%.

**Figure 6:**
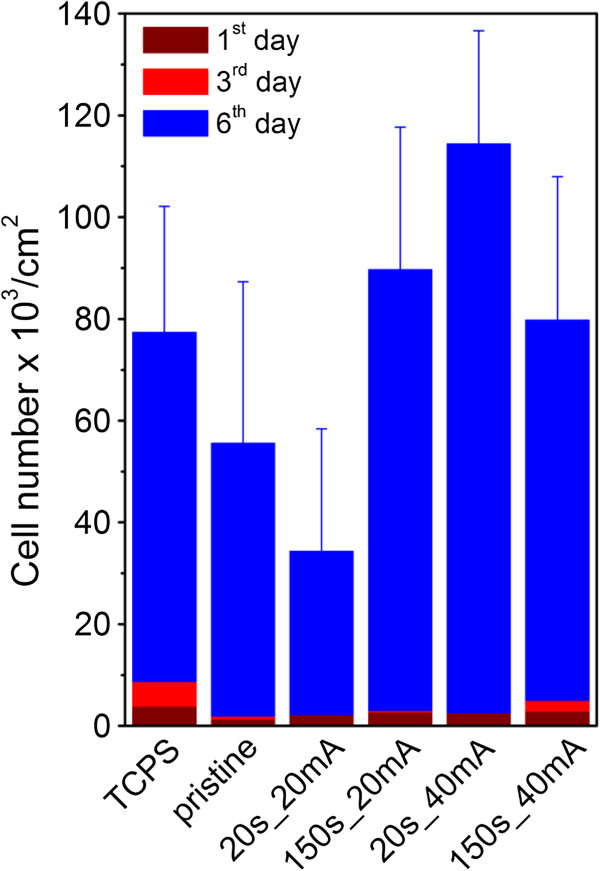
**The number of VSMCs after different cultivation times (1st, 3rd, and 6th day).** On pristine glass, gold-coated glass (20 and 150 s sputtering times and 20 mA discharge current),and gold-coated glass (20 and 150 s sputtering times and 40 mA discharge current). The number of VSMCs on TCPS was used as a standard.

The interaction of VSMCs with gold-sputtered glass substrate was studied by using a microscope. The number of adhered and proliferated cells just after seeding is shown in Figure 6. For comparison in a control experiment, the cells were also seeded, under the same conditions, on standard tissue polystyrene (TCPS). On the 1st and 3rd day after the seeding, the number of adhered cells on the pristine glass and glass/gold substrates was minimal especially in comparison with TCPS. On the 6th day (cells proliferation) after the seeding, the number of cells on pristine and gold-coated glass increases dramatically. The cell growth on pristine glass is slower than that on the TCPS. Gold coating results in dramatic increase in VSMCs proliferation, which is higher than that on the TCPS. Analogous increase in cell proliferation was observed also on substrates which are chemically grafted with gold nanoparticles [9]. From *in vitro* experiments, the viability of VSMC cells was determined to be 60% and 95% after 1 and 6 days after seeding, respectively.

**Figure 7:**
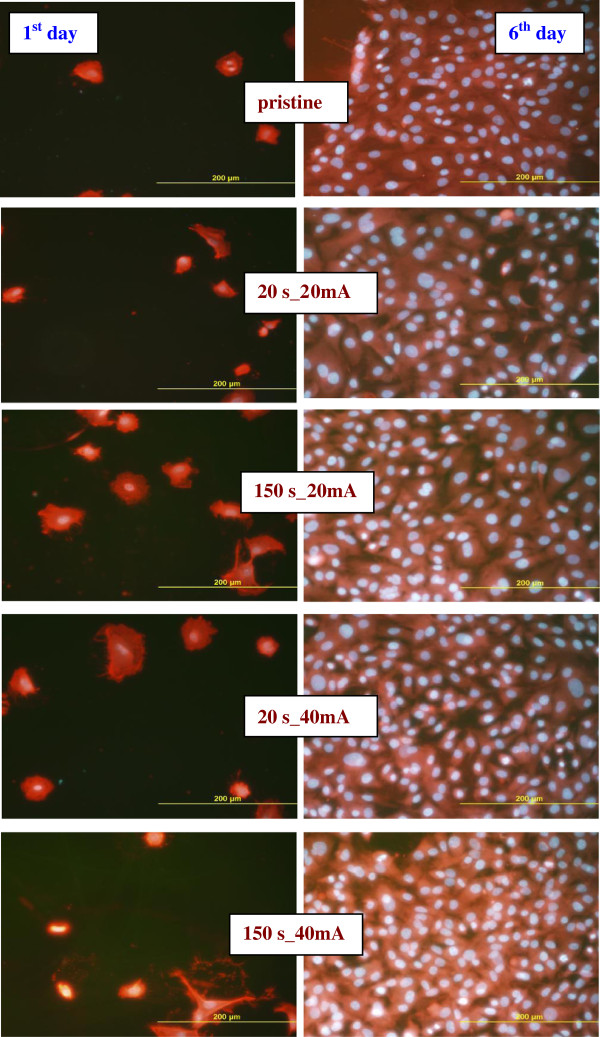
**The photographs of VSMCs adhered (1st day after seeding) and proliferated (6th day after seeding).** On pristine glass and gold-coated glass (20 and 150 s sputtering times, 20 and 40 mA discharge currents).

The cell growth is illustrated on the photographs of VSMCs adhered (1st day) and proliferated (6th day) on pristine glass and glass sputtered with gold for 20 and 150 s at discharge currents 20 and 40 mA which are shown in Figure 7. First day after the cell seeding, no significant differences in the cell size, cell form, and density between different substrates are observed. In comparison with pristine glass, the cells on the gold-coated samples exhibit higher homogeneity and better spreading of cells. Worse homogeneity of VSMCs growth and lower cell density (see Figure 6) are observed on the sample sputtered at 40 mA for 150 s. Maximum number of cells is observed on the sample sputtered for 20s at 40 mA, exhibiting lowest contact angle (see Figure 3), low surface roughness (see Table 1), and mild increase in electrical conductivity (see Figure 2). All these factors are known to facilitate VSMCs proliferation [9,19,27].

## Conclusions

Glass substrates sputtered with gold for different sputtering times and at different discharge currents were studied. The thickness of the deposited gold film is an increasing function of the sputtering time and the discharge current. Linear dependence between the sputtering time and the layer thickness is evident even in the initiatory stage of nanoparticles/layer growth. A rapid decline of the sheet resistance is observed on gold films deposited for the times above 100 s. The contact angle is a slowly increasing function of the sputtering time for discharge currents from 10 to 30 mA. After the formation of continuous gold coverage, the samples exhibit hydrophobic character. The UV–vis absorbance of gold films increase with increasing sputtering time and discharge current and film thickness. Gold deposition leads to dramatic changes in the surface morphology and roughness in comparison to pristine glass substrate. AFM images prove the creation of separated gold islands in initial deposition phase and a continuous gold coverage for longer deposition times. Gold deposition has a positive effect on the proliferation of vascular smooth muscle cells. The largest number of cells was observed on sample sputtered with gold for 20 s and at the discharge current of 40 mA. This sample exhibits lowest contact angle, low relative roughness, and only mild increase of electrical conductivity. Under the present experimental conditions, the specific contribution of individual factors to cell interaction with the substrate cannot be classified separately. The gold/glass structures studied in this work could find an application as biosensors.

## Competing interests

The authors declare that they have no competing interests.

## Authors’ contributions

AR carried out the AFM analysis, evaluated the surface morphology and roughness, and wrote and designed the study. ZN analyzed the electrical and optical properties, carried out gravimetry and goniometry measurements, and calculated the number of VSMCs of gold-coated glass samples. NSK performed the cytocompatibility tests. VS participated in the study coordination and paper correction. All authors read and approved the final manuscript.
